# Comparison of the use of manikins and simulated patients in a multidisciplinary *in situ* medical simulation program for healthcare professionals in the United Kingdom

**DOI:** 10.3352/jeehp.2021.18.8

**Published:** 2021-04-20

**Authors:** Marrit Meerdink, Joshua Khan

**Affiliations:** Bristol Medical Simulation Centre, University Hospitals of Bristol and Weston NHS Foundation Trust, Bristol, UK; Hallym University, Korea

**Keywords:** Learning, Manikins, Simulation training, Surveys and questionnaires, United Kingdom

## Abstract

**Purpose:**

Simulation training is increasingly popular in healthcare education, and often relies on specially designed manikins. However, it is also possible to work with actors, or simulated patients (SPs), which may provide a greater sense of realism. This study aimed to compare these 2 approaches, to ascertain which makes healthcare professionals feel most comfortable, which leads to the greatest improvement in confidence, and which is most beneficial to learning.

**Methods:**

This study was embedded in a pre-existing multidisciplinary in situ simulation program. A multidisciplinary group of learners from a range of backgrounds—including nurses, doctors, and other allied health professionals—were asked to complete a questionnaire about their learning preferences. We collected 204 responses from 40 simulation sessions over 4 months, from September to December 2019. Of these 204 responses, 123 described using an SP and 81 described using a manikin.

**Results:**

We found that 58% of respondents believed they would feel more comfortable working with an actor, while 17% would feel more comfortable using a manikin. Learners who used both modalities reported a significant increase in confidence (P<0.0001 for both). Participants felt that both modalities were beneficial to learning, but SPs provided significantly more benefits to learning than manikins (P<0.0001). The most common reason favoring SP-based simulation was the greater realism.

**Conclusion:**

In scenarios that could reasonably be provided using either modality, we suggest that educators should give greater consideration to using SP-based simulation.

## Introduction

### Background/rationale

Simulation is a popular approach in medical education and is often provided using manikins with various levels of fidelity. Although manikin-based simulation education has been shown to be effective [1], the technology is expensive and requires experienced operators [2]. In our experience, working in simulation education in the United Kingdom, simulation has become synonymous with manikin-based training. The use of manikins has a number of benefits, giving learners the opportunity to practice clinical and communication skills without fears of harming or upsetting a real or simulated patient (SP). However, our participants have reported difficulties engaging with a manikin as if it is a real patient. This can be a source of fixation, hindering participants’ ability to fully engage in the learning. To address these concerns, we have started working with actors, or SPs, who behave like real patients and provide human interaction. Evidence shows this approach to be effective in training for not only communication skills [3], but also the management of clinical emergencies [4]. Additionally, healthcare workers behave more similarly when faced with SPs to how they would in real clinical situations [5].

Although some topics are especially suited to a certain approach, many scenarios can, with minor adjustments, be delivered using either an SP or a manikin. Thus far, limited research has explored which option is best in such situations, with previous studies only analyzing specific clinical situations or settings [2,6], and including small sample sizes [5,7]. Hence, we conducted a comparative study to investigate which modality participants responded better to in scenarios that can be run using either an SP or a manikin, in various clinical settings.

### Objectives

It aimed to ascertain which approach (an SP or a high-fidelity manikin) makes healthcare professionals feel most comfortable, which leads to the greatest improvement in confidence, and which is most beneficial to learning through simulation.

## Methods

### Ethics statement

This project was embedded in an established simulation program. Participants answered additional questions as part of the usual feedback questionnaire after each session. No changes were made to the teaching. The safety of the learners was ensured throughout, and there was no potential harm to participants. There was an option for learners to observe rather than actively participate. Informed consent was obtained before each session and before completing the questionnaire. We sought advice from the organization’s Research Information Officer and information from the United Kingdom National Health Service Health Research Authority. We were advised that there was no need to obtain further formal ethical approval.

### Study design

This was a survey-based observational study.

### Setting

This study was embedded in the Bristol Medical Simulation Centre’s regular multidisciplinary *in-situ* simulation program. Over 4 months, from September to December 2019, scenarios were delivered with an SP (a faculty member briefed in detail about their role) or a manikin (Laerdal’s SimMan Essential or Gaumard’s Hal S23201). Scenarios specifically requiring a manikin, such as cardiac arrests, or those clearly more suited to an SP, such as communication-based mental health scenarios, were excluded. Both the manikins used have a range of high-fidelity features including palpable pulses, audible heart and breath sounds, eye movements, pupil movements, and monitors displaying vital signs, all of which can be manipulated by the facilitator.

The following scenarios were included: anaphylaxis, asthma exacerbation, chronic obstructive pulmonary disease exacerbation, extravasation of chemotherapy, fall, hypoglycemia, preparation for intubation, intracranial bleed, major hemorrhage, pulmonary embolism, seizure, sepsis, transfusion reaction, and an unresponsive patient. After each scenario and debriefing session, participants completed a confidential questionnaire.

### Participants

The project was carried out at the University Hospitals of Bristol and Weston, a large tertiary teaching hospital in the United Kingdom, operated by the National Health Service. The organization comprises a general acute hospital including inpatient and outpatient medical and surgical care, a specialized heart institute, an eye hospital, a dental hospital, and a hematology and oncology center, as well as a peripheral community rehabilitation hospital at a separate site. Participation was voluntary and encouraged as part of the organization’s ongoing multidisciplinary simulation program. This program provides regular simulation training provided in the learners’ usual clinical areas, which are accessible to all staff members across the hospital, including students. All the learners participating in this training program during the study period, between September and December 2019, were invited to participate in this project. None of those invited declined to take part, resulting in a wide and varied sample of participants.

### Variables

There were 3 main variables. The first was learner preferences, for which we compared the learner’s self-reported change in confidence from before the scenario to afterwards to assess the difference between those who used an SP and those who used a manikin. The second was learners’ comfort, where we measured which approach learners believed would make them feel more comfortable in a scenario. The third was benefit to learning, where we asked which approach learners believed to be more beneficial to their learning, an SP or a manikin. We also compared other variables including the level of content, relevance to practice, importance, enjoyment, and regularity.

### Data source/measurement

Data were participants’ responses to questionnaires. The first part of the questionnaires consisted of the place of simulation teaching and job type. The next 5 variables (topics) were as follows: appropriateness of level of content, relevance of topic, importance of topic, enjoyment of simulation, and preferred regularity. Two items elicited information on participants’ confidence level before and after the simulation training. Participants were also asked about the benefits of actors and manikins. We used the term “actor” to describe the SP in the questionnaire, as this is a commonly used term that did not require further explanation (Supplement 1).

### Validity and reliability testing of the measurement tool (questionnaire)

Content validity was established by discussion among the authors. Two authors made a questionnaire for this comparison study. All items’ content was related to the medical simulation program. The reliability of the 5 variables (the level of content, relevance to practice, importance, enjoyment, and regularity) was significant (Cronbach α=0.9928, degrees of freedom=244, P<0.05). Response data are available from Dataset 1. Data for the comparison test and reliability test are available from Dataset 2.

### Study size

During the 4-month study period, all consecutive learners in the organization’s regular multidisciplinary simulation program were invited to participate in the study. *Post hoc* power analysis was carried out to estimate the power with a given sample size using the G*Power tool (Heinrich-Heine-Universität Düsseldorf, Düsseldorf, Germany; http://www.gpower.hhu.de/) [8], which showed that the comparison study for participants’ confidence level before and after the simulation training and the benefits of actors and manikins was adequately powered for tests resulting in statistically significant findings. For the matched-pairs Wilcoxon signed-rank test for the actor group’s confidence before and after the simulation training, the power was 1.0. The input values were as follows: 2 tails; parent distribution, Laplace; effect size, 1.4077; alpha error probability, 0.05; and total sample size, 123. For the same test for the manikin group’s confidence before and after the simulation training, the power was 1.0. The input values were as follows: 2 tails; parent distribution, Laplace; effect size, 1.7714; alpha error probability, 0.05; and total sample size, 81. For the comparison of benefits between actor and manikin use, the power was 1.0. The input values were as follows: 2 tails; parent distribution, Laplace; effect size, 1.8868; alpha error probability, 0.05; and total sample size, 204.

### Statistical methods

Data were compiled from the questionnaire. Where a Likert scale was used, an equivalent ranking of 1 to 5 was assigned depending on the rating (strongly disagree=1 to strongly agree=5). Participants were asked to justify their answers. In the statistical analyses, P<0.05 was deemed a significant value. Self-reported scores for confidence before and after simulation-based training were compared using the 2-tailed Wilcoxon signed-rank test to assess whether there was a change in confidence in assessing and managing the patient in the scenario. Participants were also asked how beneficial they found the simulation modalities to be to their learning. The scores given for manikin- and SP-based scenarios were compared, again using the 2-tailed Wilcoxon signed-rank test. The Mann-Whitney U-test was used to compare the change in confidence between the 2 groups, and to compare the enjoyment of the session between those who participated in manikin- and SP-based simulations.

## Results

### Participants

We collected 204 responses from 40 scenarios over 4 months. Of these, 123 used an SP and 81 used a manikin. Our participants comprised professionals from a range of backgrounds. Their roles are presented in Table 1. They were split over several sites. Of the 204 participants, 47 worked in a peripheral community hospital, with 11 of those working in the Day Surgery and Endoscopy Unit, 10 in the Dentistry Unit, and the remaining 26 in inpatient rehabilitation wards. The remaining 157 participants worked in a large city-center hospital complex, with 24 working in outpatient areas, 13 in a specialist eye hospital, 18 in surgical inpatient wards, 4 in the endoscopy unit, and the remainder in various medical inpatient wards.

### Learner preferences

The majority (n=119, 58.3%), of respondents stated that they would feel more comfortable working with an actor rather than a manikin in simulations. Fifty respondents (24.5%) had no preference, and 35 respondents (17.2%) indicated that they would feel more comfortable using a manikin (Supplement 1).

### Learner confidence

Using the Wilcoxon signed-rank test, we found a significant increase in confidence ratings in both those who worked with an SP (P<0.0001) and those who used a manikin (P<0.0001). The mean confidence rating for the SP cohort was 2.85 (median=3.00) before the training, increasing to 3.90 (median=4.00) after the training. For the manikin cohort, the mean was 3.14 (median=3.00) pre-training, which increased to 3.96 (median=4.00) post-training.

As confidence ratings improved in both groups, we used the Mann-Whitney U-test to compare the increase in confidence between the simulation approaches. The mean increase in confidence working with an SP was +1.05, whilst the mean increase in confidence when using a manikin was +0.83. The difference in confidence change was not significant (P=0.0536).

### Benefit to learning

Using the Likert scale described above, participants reported a mean benefit to learning from manikin-based simulation of 2.97 (median=3.00). In contrast, the mean reported benefit from SP-based training was 4.17 (median=4.00). Using the Wilcoxon signed-rank test to compare these results, actors were believed to provide significantly more benefit to learning (P<0.0001). When asked why actors were believed to be more beneficial to their learning, the most common response was that using actors felt “more realistic.” The second most common reason was that it was better for “communication” and that there were “verbal and non-verbal” cues. The common reasons for preferring a manikin included that it felt “less pressured” and that participants had no fear of hurting the manikin.

### Level of content, relevance to practice, importance, enjoyment, and regularity

Comparisons between the actor and manikin groups were conducted using the Mann-Whitney U-test. For level of content, there was no difference (P=1.0). Relevance to clinical practice was not significantly different (P=0.1765). The importance of the topic was not significantly different (P=0.4856). Likewise, no significant differences were found for enjoyment during the simulation learning (P=0.4355) or the regularity of simulation training (P=0.4959).

## Discussion

### Key results

Our results show that learners not only believed that working with an SP would make them more comfortable, but also that SP-based simulation would be more beneficial to their learning than manikin-based simulation. This is an essential finding, as manikin use is pervasive in simulations. These findings, combined with the high costs associated with purchasing manikins and the requirement for trained operators, suggest that SP-based simulation should take precedence over manikin-based simulation in certain settings.

### Interpretation

The most common reason why participants believed SP-based simulation to be more beneficial was that it is more realistic, as it was more intuitive to interact with a person than with a manikin. Additionally, participants felt working with SPs was “better for communication.” Indeed, simulation is uniquely suited to training related to non-technical competencies and human factors. As a participant commented, it is “helpful to have a person giving verbal and non-verbal responses.” This heightened sense of reality and interpersonal communication makes SP-based simulation especially useful for training on human factors. The creation of a psychologically safe environment is crucial in simulation, as it allows learners to make mistakes, ask questions, and engage in self-correcting behaviors [9]. We have shown that learners were more comfortable working with SPs, suggesting that SP-based simulation creates a more psychologically safe space. This is a significant factor in favor of SP-based simulation.

Despite these advantages, working with SPs is not without challenges. For one, the SP must be clearly briefed on the expected behaviors. Additionally, learners should be clear on what information they should gain from the SP, and what they should find from other sources such as technology or the facilitator. For the SP’s physical and psychological safety, there must also be clear instructions for learners about what actions and procedures should and should not be carried out on the SP, and the SP and the facilitator must be able to communicate throughout the scenario to ensure this. More detailed guidance on best practice standards has been published previously [10] and is outside the scope of this article. Although these are factors to consider when running SP-based scenarios, they can be easily managed, and do not constitute barriers to its use.

Most importantly, as discussed, not all scenarios can be flexibly provided using either approach; the choice of modality must depend on the case being simulated [2]. We are by no means arguing that SPs should completely replace manikin-based simulation. Educators should consider the advantages and disadvantages of both modalities and choose the most appropriate approach on a case-by-case basis. We do, however, suggest that educators should be mindful of the above results; learners believe that SPs make them feel more comfortable and are more beneficial to their learning. Hence, where either modality is a realistic option, the use of SPs should be encouraged.

### Limitations

Of course, there are limitations to our methods. First, self-rated Likert scales were used due to their ease of use for participants and to maintain consistency for comparison to other simulation programs at the same center. However, there are inherent problems in using this method, especially in terms of subjectivity and variability in how participants may interpret the scale. Additionally, although the running of the scenarios was kept as similar as possible, there was some unavoidable variability in how the scenarios were provided. As the simulation was delivered as part of an established teaching program, the facilitators’ central focus was on providing the best educational experience for the learners, rather than maintaining perfect conditions for comparison. An example of this variation is that the training was mostly delivered in each learner’s normal ward environment. This was beneficial as it facilitated the participation of a wide variety of multidisciplinary staff in a familiar environment, but it also led to the need to make subtle changes to the scenarios to fit specific clinical areas. Efforts were made to minimize this variability, including using the same staff to facilitate scenarios and using the same scenario scripts in both arms.

Thus, although our results favor the use of more SP-based simulations, these limitations mean that more definitive evidence, with controlled comparisons and objective outcome measures, would be needed to provide more conclusive evidence.

### Conclusion

These results suggest that, despite the ubiquity of manikin-based scenarios in simulation, learners believe that SP-based simulation makes them more comfortable and is more beneficial to their learning. As discussed, certain scenarios are clearly suited to one or the other approach. However, in scenarios that could be reasonably carried out using either modality, we encourage educators to give greater consideration to using SP-based simulation.

## Figures and Tables

**Table 1.** Participants’ professional groups and the type of simulation in which they participated

Professional group	No. of participants	SP-based simulation	Manikin-based simulation
Registered nurses	91	51	40
Student nurses	35	20	15
Nursing assistants	33	16	17
Doctors	13	5	8
Occupational therapists	12	12	0
Dental nurses	4	4	0
Dentists	3	3	0
Pharmacists	3	3	0
Speech and language therapists	2	2	0
Dental students	2	2	0
Plaster technicians	2	2	0
Trainee nursing assistants	2	2	0
Physician’s associates	1	0	1
Therapy technicians	1	1	0
Total	204	123	81

## Footnotes

**Data availability** Data files are available from Harvard Dataverse: https://doi.org/10.7910/DVN/OZZIOM Dataset 1. Raw response data of the participants to the questionnaire after each simulation session. Dataset 2. Data table for the comparison analysis.

## Supplementary materials

Supplementary files are available from Harvard Dataverse: https://doi.org/10.7910/DVN/OZZIOM

Supplement 1. Feedback questionnaire completed by participants after the simulation session.

Supplement 2. Audio recording of the abstract.
