# Supplementary material for: Comparison of the use of manikins and simulated patients in a multidisciplinary in situ medical simulation program for healthcare professionals in the United Kingdom
Source: J Educ Eval Health Prof. 2021 Apr 20;18:8. doi: 10.3352/jeehp.2021.18.8 (PMC8175761; doi:10.3352/jeehp.2021.18.8)
Supplement: Supplementary file 3 — Supplement 1. Feedback questionnaire completed by participants after the simulation session. [file jeehp-18-08-suppl.docx]

**Supplement 1. Feedback questionnaire completed by participants after simulation session**

Please note a number of questions not specifically related to this project but required by the organisation were also included in the questionnaire.

Actor [ ] Manikin [ ]

**Simulation Training Feedback**

On which ward did you receive the simulation teaching?

|  |
| --- |

What is your role *(OPTIONAL)*

| Nursing Assistant | Registered Nurse | Doctor | Other……. |
| --- | --- | --- | --- |

The content was at an appropriate level *(please circle)*

| Strongly Disagree | Disagree | Neutral | Agree | Strongly Agree |
| --- | --- | --- | --- | --- |

The simulated scenario was relevant to my clinical practice *(please circle)*

| Strongly Disagree | Disagree | Neutral | Agree | Strongly Agree |
| --- | --- | --- | --- | --- |

How did you rate your confidence at **ASSESSING** and/or **MANAGING** this type of patient **BEFORE** the simulation teaching *(1 = not confident and 5 = very confident)*

| 1 | 2 | 3 | 4 | 5 |
| --- | --- | --- | --- | --- |

How did you rate your confidence at **ASSESSING** and/or **MANAGING** this type of patient **AFTER** the simulation teaching *(1 = not confident and 5 = very confident)*

| 1 | 2 | 3 | 4 | 5 |
| --- | --- | --- | --- | --- |

I feel that important topics were addressed in the debrief/teaching *(please circle)*

| Strongly Disagree | Disagree | Neutral | Agree | Strongly Agree |
| --- | --- | --- | --- | --- |

I enjoyed the session *(please circle)*

| Strongly Disagree | Disagree | Neutral | Agree | Strongly Agree |
| --- | --- | --- | --- | --- |

Simulation should be a regular part of ward based training *(please circle)*

| Strongly Disagree | Disagree | Neutral | Agree | Strongly Agree |
| --- | --- | --- | --- | --- |

Which do you believe would make you feel more comfortable during the scenario?

*(please circle)*

| Actor | Manikin | No Opinion |
| --- | --- | --- |

Using an **ACTOR** would be most beneficial to my learning in this scenario *(please circle)*

| Strongly Disagree | Disagree | Neutral | Agree | Strongly Agree |
| --- | --- | --- | --- | --- |
| *Reason for response:* | | | | |

Using a **MANIKIN** would be most beneficial to my learning in this scenario *(please circle)*

| Strongly Disagree | Disagree | Neutral | Agree | Strongly Agree |
| --- | --- | --- | --- | --- |
| *Reason for response:* | | | | |

Participating in this teaching session has contributed to me feeling **VALUED** as a member of UHBT staff *(please circle)*

| Strongly Disagree | Disagree | Neutral | Agree | Strongly Agree |
| --- | --- | --- | --- | --- |
| *Reason for response:* | | | | |

| Is there anything that you found particularly useful? |
| --- |
| How could we improve this teaching session? Please list some scenarios you would like to see in the future |
| Please list two key lessons/take home messages from today that you will aim to apply to your future clinical practice |
